# Temporal changes of serum cytokine/chemokine levels in patients of Nakajo-Nishimra syndrome treated with tocilizumab

**DOI:** 10.1186/1546-0096-13-S1-P169

**Published:** 2015-09-28

**Authors:** N Kanazawa, Y Nakatani, Y Inaba, K Kunimoto, F Furukawa, F Ozaki

**Affiliations:** 1 Wakayama Medical University, Department of Dermatology, Wakayama, Japan; 2 Kyoto University, Center for iPS Cell Research and Application, Kyoto, Japan

## 

In Nakajo-Nishimura syndrome (NNS), proteasome disability due to a loss-of-function *PSMB8* mutation induces storage of ubiquitinated proteins and overproduction of inflammatory cytokines and chemokines. However, the precise mechanisms causing complex phenotypes of the disease, including pernio-like eruptions, lipodystrophy and calcification of basal ganglia, is mostly unclear. As IL-6 overproduction in association with p38 hyperactivation was supposed to have a role in NNS (Arima *et al*, PNAS 2011), tocilizumab, a monoclonal antibody for IL-6 receptor, has recently been applied for two patients with NNS after informed consents were obtained. Decreased serum CRP and CPK levels in both patients and improved myalgia and arthralgia in one patient have been observed, whereas none of decrease in serum LDH level or improvement of fever and eruptions have been achieved. By analysis of serum cytokine/chemokine levels, IL-6, G-CSF and MCP-1 levels have changed in accordance to the CRP level, whereas IP-10 has shown constantly high levels independent of the CRP level. Furthermore, both the patients-derived peripheral blood monocytes and monocytes differentiated from a patient-derived iPS cells produced higher level of IP-10 than control cells after IFNgamma stimulation. These findings suggest that monocyte-derived IP-10 has a major role in pathogenesis of the sustained/progressing phenotypes in NNS.

